# Trauma-and-Violence-Informed Care for Victim-Survivors of Domestic, Family and Sexual Violence: A Qualitative Meta-Synthesis of Service Providers’ Perspectives

**DOI:** 10.1177/15248380251383933

**Published:** 2025-11-04

**Authors:** Mia Davies, Lata Satyen, John W. Toumbourou

**Affiliations:** 1 Deakin University, Burwood, VIC, Australia

**Keywords:** domestic violence, family violence, sexual violence, help seeking/reporting/disclosure, treatment/intervention, practitioners, trauma-informed care

## Abstract

Formal support is essential for promoting recovery and healing of victim-survivors of domestic, family, and sexual violence (DFSV). Trauma-and-violence-informed care (TVIC) aims to improve outcomes and minimize harm for trauma survivors accessing support. Despite its existence, services are not adequately meeting the needs of victim-survivors of DFSV and continue to cause harm. Guiding frameworks that can be consistently applied across services are thus needed. This qualitative meta-synthesis systematically integrates service providers’ perspectives on what constitutes effective TVIC for victim-survivors of DFSV to determine practices and principles to better support victim-survivor recovery when providing care. Seven databases were searched for peer-reviewed articles and gray literature in June 2023, with an updated search in September 2024. Twenty-nine articles were included in the study, representing 694 service providers across a range of settings. A thematic synthesis approach resulted in eight key themes outlining principles of TVIC for DFSV service provision, including six individual-level themes: (a) safety, (b) survivor-centered care, (c) respectful emotional support, (d) non-judgmental, reciprocal client-practitioner relationship, (e) supporting victim-survivors to thrive, and (f) cultural safety and inclusivity; and two system-level themes: (g) provision of effective and accessible services, and (h) providing holistic services. The findings identified a holistic, trauma-and-violence-informed, survivor-centered, and inclusive approach to supporting victim-survivors, describing principles, values, and specific practices which can be applied by practitioners and services to improve service outcomes and facilitate recovery and healing.

## Introduction

Domestic and family violence (DFV) refers to violent, threatening, or controlling behaviors perpetrated by a current or former intimate partner or other family member, that are either sexual, psychological, physical, emotional, spiritual, technological, or financial in nature (United Nations, n.d.); sexual violence refers to “any sexual act, attempt to obtain a sexual act, or other act directed against a person’s sexuality using coercion, by any person regardless of their relationship to the victim, in any setting” (World Health Organization [WHO], 2024). Domestic, family, and sexual violence (DFSV) encompasses these forms of violence and is recognized by the World Health Organization as a form of violence against women. DFSV is considered a global public health crisis due to its high prevalence (WHO, 2024), with one in three women worldwide experiencing DFSV in their lifetime (Sardinha et al., 2022). The effects of experiencing DFSV are pervasive, long-lasting, and detrimental to health and general functioning, including long-term negative impacts to physical health (Dillon et al., 2013), significantly poorer mental health outcomes (Coker et al., 2002; Dillon et al., 2013; Woods et al., 2008), and social difficulties, including stigmatization and economic and housing insecurity (Baker et al., 2010; Murvartian et al., 2023). Supporting the health and well-being of those affected by DFSV is necessary to mitigate its detrimental impacts, with further research and action required to address this highly significant gendered, global issue.

Recovery after DFSV is a continuous process during which victim-survivors rebuild their lives and undertake healing of their physical and mental health in the aftermath of DFSV, conceptualized as a non-linear, multidimensional, and complex journey that is unique to each victim-survivor (Carman et al., 2023; D’Amore et al., 2021; Flasch, 2020). Research has indicated that victim-survivors can experience significant reductions in symptoms of depression, anxiety, and post-traumatic stress disorder (PTSD) over time (Blasco-Ros et al., 2010; Micklitz et al., 2024). However, recovery involves not only mitigating the detrimental health and well-being effects of DFSV, but “mov[ing] forward. . .to achieve positive, satisfying lives and relationships, as well as optimal functioning in various areas of their lives” (Flasch et al., 2017, p. 3375). Flasch et al. (2017) described a recovery framework consisting of various intrapersonal and interpersonal processes, including those related to identity, freedom, healing, acceptance and forgiveness, and understanding and developing healthy relationships. Additional research has identified four key components of recovery consistent across victim-survivor accounts: survival and safety, gaining freedom (i.e., regaining control of their lives), moving on (i.e., healing and wellbeing), and a better life (i.e., finding enjoyment and purpose in life; Carman et al., 2023). Recovery is thus a crucial and transformative process.

Engagement with formal support is an essential factor in facilitating recovery after DFSV. Formal support refers to the various services that support victim-survivors, including legal, advocacy, healthcare, social, and crisis intervention services, providing victim-survivors with crucial resources including medical care, counseling, financial assistance, housing, and legal support (Coker et al., 2000). Many victim-survivors access a range of services and consequently report improved decision-making, safety, self-efficacy, and coping abilities (Bennett et al., 2004). Research indicates improvements in anxiety, depression, and PTSD symptoms in victim-survivors of DFSV after psychological support (Hameed et al., 2020; Micklitz et al., 2024). Despite these benefits, rates of accessing supports vary considerably globally based on factors including community, culture, and identity. Victim-survivors from culturally diverse groups, immigrant or refugee populations, and LGBTIQA+ individuals face additional barriers to support, such as systemic discrimination, mistrust in services, lack of cultural sensitivity, and language barriers (Calton et al., 2015; Green et al., 2024; Lowe et al., 2026). Help-seeking rates are particularly low in low- and middle-income countries (Stiller et al., 2025).

Important considerations need to be made when supporting victim-survivors of domestic abuse. The trauma resulting from DFSV is particularly complex and distinct from that of non-interpersonal traumas (e.g., natural disasters) due to the betrayal involved when violence is perpetrated by a trusted person (Martin et al., 2013) and the often prolonged, repeated nature of the traumatic experience. Due to the complex emotional and psychological effects of DFSV, victim-survivors are at risk of being retraumatized when accessing services (Dallam, 2010; Duckworth & Follette, 2012). Furthermore, the effects of DFSV are far-reaching and victim-survivors access a range of resources and pathways for support across many areas, with domestic abuse tending to intersect with other experiences of marginalization and oppression, such as homelessness, racism, and poverty (Imkaan, 2019). Victim-survivors of DFSV thus require unique and tailored supports with specific service provision requirements.

Given these unique considerations, a framework guiding the implementation of effective practices that is consistently applied across DFSV services is necessary to promote victim-survivor recovery. Trauma-informed care (TIC) is a service provision framework aimed at improving recovery outcomes and reducing the risk of retraumatization for service users through sensitivity to the impacts of trauma (Substance Abuse and Mental Health Services Administration [SAMHSA], 2017). TIC seeks to meet the priorities, preferences, and goals of trauma survivors by promoting safety, choice, and control across all levels of service delivery (Liu et al., 2024). TIC is distinct from trauma-specific interventions as it relates to the approach taken when supporting victim-survivors and can be utilized across interventions, whereas trauma-specific interventions focus on addressing trauma symptoms. SAMHSA identified the following six foundational principles for TIC: empowerment, voice, and choice; peer support; collaboration and mutuality; trustworthiness and transparency; cultural, historical, and gender issues; and safety (SAMHSA, 2014). TIC has been associated with better psychological health, well-being, and recovery for service users and better service engagement (Chu et al., 2024; Gilbert et al., 2012; Liu et al., 2024).

TIC has been extended to acknowledge the structural and contextual factors associated with violence perpetration and victimization through the concept of trauma-and-violence-informed care (TVIC; Wathen & Mantler, 2022). Through TVIC, conceptualizations of violence are expanded from focusing on the individual to considering the broader contexts in which violence occurs, considering factors such as legislation, economic and social inequities, cultural norms associated with violence, and systemic and historical violence. TVIC is guided by four principles: (a) understanding and awareness of trauma and violence and their impacts; (b) prioritizing physical, emotional, and cultural safety; (c) promoting person-centered collaboration and choice; and (d) incorporating a strengths-based approach (Wathen & Varcoe, 2023). TVIC recognizes the importance of an equity-based approach incorporating intersectionality theory, which describes the interaction between social, political, cultural, and historical dimensions that create systems of power and oppression (Crenshaw, 1991). TVIC is particularly important when working with victim-survivors from diverse cultural backgrounds, sexuality, gender, immigration, and housing statuses. The principles of TVIC have not yet been consistently adapted to the unique requirements for supporting victim-survivors of DFSV.

Previous research has explored the principles of TIC in the DFSV sector, healthcare professionals’ applications, and victim-survivor expectations for care. Wilson et al. (2015) reviewed TIC principles across DFSV organizations, identifying six themes: promoting emotional safety, restoring choice and control, facilitating connection, supporting coping, responding to identity and context, and building strengths. The findings emphasized the importance of safe, supportive, and inclusive practices and a non-judgmental approach that promotes victim-survivor agency. A recent review of healthcare professionals’ experiences applying TIC with DFV victim-survivors demonstrated that medical personnel avoid pathologizing, advocate for victim-survivors’ preferences and priorities, and aim to build trusting relationships with patients through non-judgmental communication, validation, and providing space to be heard (Bulford et al., 2024). Research into victim-survivors’ perspectives demonstrated that they value the creation of safe, non-judgmental, and respectful spaces, choice, sensitivity to gender and cultural issues, and the development of trusting and collaborative relationships (Liu et al., 2024). Supporting these findings, Tarzia et al. (2020) reviewed victim-survivors’ expectations from healthcare professionals following DFV disclosure, identifying kindness and care, validation and understanding, providing helpful practical support, and choice and control as essential components.

Despite this research and the existence of TVIC, research findings from mainly high-income countries report numerous instances of services not adequately supporting victim-survivors and engaging in practices that are harmful to their recovery. Victim-survivors have described experiencing denial and dismissal, misdiagnosis, discrimination, blame, and stigmatization when accessing services (Kennedy et al., 2023; Murvartian et al., 2023; Taylor, 2020). The dynamics of coercion and control can be recreated for victim-survivors when they are without agency and providers are perceived as controlling (Trevillion et al., 2016). Service accessibility is lacking, particularly for victim-survivors from differing cultural and socioeconomic backgrounds and abilities (Kennedy et al., 2023; Milaney et al., 2019). Furthermore, inadequacies such as services’ lack of cultural understanding and relevance deter victim-survivors from seeking help (Green et al., 2024).

Equitable, intersectional, and culturally responsive approaches are required to mitigate harms and increase accessibility (Ravi et al., 2022). Furthermore, implementation of TVIC is hindered by resource constraints, inadequate investment in training, demand pressure, disparate services, and insufficient organizational commitment (Emsley et al., 2022; Wathen & Mantler, 2022). Guiding frameworks for the application of effective TVIC across the spectrum of DFSV services are limited, thus, effective ways to tailor and apply TVIC to the specific requirements of supporting DFSV victim-survivors are needed.

There has yet been no investigation into the perspectives of service providers on what constitutes effective support for victim-survivors across the range of supports and services in the DFSV sector (i.e., the sector encompassing all services that victim-survivors access to seek support, contributing to their recovery and well-being). Service providers work with many diverse victim-survivors across stages and settings, with extensive, first-hand insight into service implementation, the operationalization of TVIC, and system-level considerations. Qualitative research allows researchers to capture, understand, and describe complex phenomena (Sofaer, 1999). A framework for supporting victim-survivors of DFSV is complex in that it encapsulates practices and approaches across various services and interactions. Thus, the current study aimed to determine what constitutes effective TVIC for victim-survivors of DFSV from the perspectives of service providers through a systematic synthesis of the qualitative literature. This review extends the literature by reviewing service provider perspectives across all service areas and by outlining a framework that encompasses a broad array of service provision types facilitating any area of recovery, from brief interactions to ongoing support.

## Method

### Search Strategy

In June 2023, with an updated search in September 2024, seven databases were searched for peer-reviewed articles. The search strategy involved the use of subject headings with specific combinations of various synonyms related to the key concepts “trauma-informed care,” “domestic violence,” “practitioners,” and “perspectives.” See Supplemental Appendix A1 for a complete list of the databases and search terms. As this is an emergent area of research, no restrictions were placed on the year of publication of studies.

### Eligibility Criteria

Studies were included in the analysis if the following criteria were met: the study investigated care/services provided for victim-survivors of DFSV; participants included at least one professional stakeholder (practitioners, volunteers, advocates, policymakers, researchers, or observers) who worked in the DFSV sector or with victim-survivors of DFSV, aged over 18 years; stakeholder’s perspectives of what practices/models constitute effective care for victim-survivors were included; primary, peer-reviewed, and published in English. Studies were excluded from the review if the above criteria were not met and if: perspectives of service providers had not been separated from those of victim-survivors in the analysis; service providers worked only with victim-survivors aged under 18 years; the care provided in the study was not related to TIC or was not considered by service providers to be the favored approach to care.

### Study Screening

Covidence (Veritas Health Innovation, 2023), a systematic review management system, was used throughout the screening process to facilitate access for all reviewers and to ensure decision-making was recorded. Using EndNote referencing software, the titles and abstracts of all studies found in the initial search were imported into Covidence, which then removed duplicates. The titles and abstracts were screened for their relevance by a single reviewer (MD). The full texts of the articles were then reviewed by six reviewers (MD, JB, JL, NO, CN, and VDB) to determine whether they met eligibility for inclusion or exclusion, with each paper reviewed by one reviewer against clearly defined inclusion and exclusion criteria. Once this process was completed, two reviewers (MD and LS) then reviewed included articles to determine whether inclusion criteria were met and to make final decisions where there was uncertainty. Figure 1 displays the Preferred Reporting Items for Systematic Reviews and Meta-Analyses (PRISMA) flow diagram demonstrating the outcomes of the study selection process.

**Figure 1.**
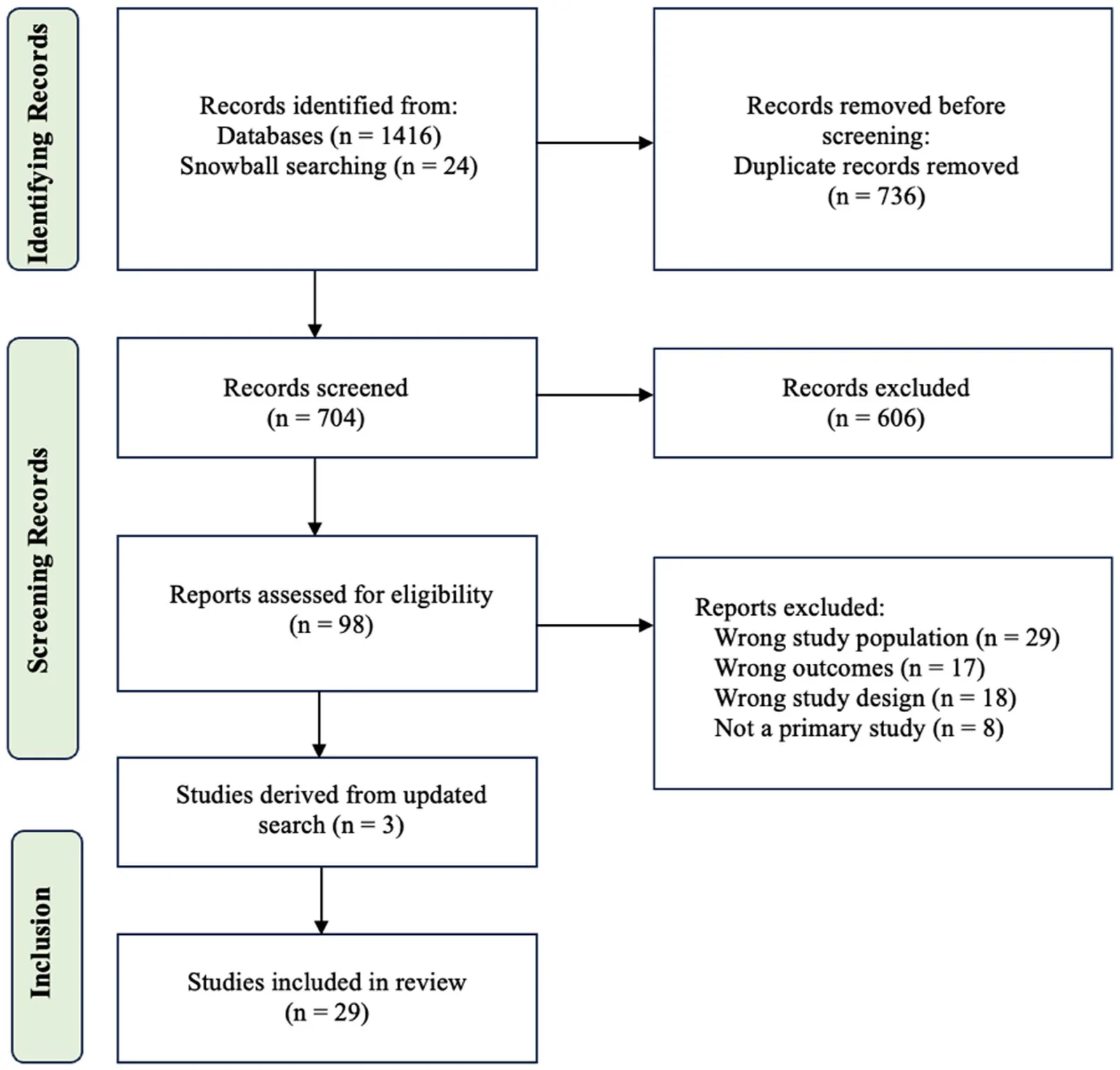
PRISMA flow diagram for study selection. *Source.* Adapted from Page et al. (2021). *Note.* PRISMA = Preferred Reporting Items for Systematic Reviews and Meta-Analyses.

### Data Extraction

Data from the included studies were extracted by a single reviewer (MD) into a Microsoft Word document with tables developed specifically for this review and then reviewed by two other reviewers (JT and LS). Separate tables were used for each included study and included relevant methodological information, such as sample characteristics, study location, and data collection methods. A table summarizing the methodological information is presented in Supplemental Appendix Table B1. Relevant data were extracted from the “Results” section of included studies for analysis. Data were deemed relevant if they were primary data pertaining to the perspectives of service providers on the provision of services to victim-survivors, including quotations from participants and interpretations by the authors.

### Quality Assessment

The Critical Appraisal Skills Program Qualitative Checklist was used to undertake a quality assessment of the included studies (Critical Appraisal Skills Programme, 2024). Quality assessment was completed by three reviewers (JT, LS, and MD), see Supplemental Appendix Table B2 for results. For each quality requirement, the reviewer selects a “Yes,” “No,” or “Can’t Tell” value. Two reviewers assessed the quality of each article. Regardless of assessed quality, all studies were included.

#### Data Analysis

Extracted data were imported into the MAXQDA qualitative data analysis software to be analyzed (VERBI Software, 2021). The thematic synthesis qualitative data analysis approach developed by Thomas and Harden (2008) was used to analyze patterns and develop themes from the data, as explained in the following sections. A reflexivity journal was kept throughout this process to document decisions and reflect upon and reduce the influence of potential biases.

### Stages 1 and 2: Coding and Developing Descriptive Themes

The first and second stages of analysis incorporated line-by-line inductive coding and the development of descriptive themes. Inductive coding is data-driven and can therefore reduce the influence of researcher bias and preconceptions (Braun and Clarke, 2006). Line-by-line coding involved the researcher (MD) inductively assigning one or more codes to each sentence, with each receiving at least one code, while most were assigned more than one. A total of 388 codes were developed. Codes were examined to check for consistency and duplicates, with additional codes added if needed. The codes were then reviewed and verified by two researchers (MD and LS) and arranged into a hierarchical tree structure, with groupings based on similarities and differences between them; 25 code groupings were identified. Eight descriptive themes were then developed to describe the meaning of layers of code groupings.

### Stage 3: Generating Analytical Themes

The third stage of the analysis involved researchers meeting to discuss the eight descriptive themes in relation to the research aim. During this discussion, analytical themes were generated from the descriptive themes. This occurred in an iterative manner until all descriptive themes were included and the aim of the research had been adequately captured by the themes. Under each theme, sub-themes that had relevance to practices were extracted and summarized.

## Results

The results, displayed in Figure 1, included 29 studies published between 2016 and 2024. The findings represented 694 service providers from various fields who worked with victim-survivors in seven countries across a range of settings; the services included DFSV shelters, hospitals, campuses, crisis hotlines, advocacy agencies, and employment, substance use, and criminal justice services. Eight themes outlined the core elements of effective TVIC from the perspectives of service providers when providing support to victim-survivors of DFSV, including principles, approaches, and specific practices (see Supplemental Appendix Table B3 for a detailed summary). Six themes were identified at the individual level, relating to practices and values to be incorporated into service providers’ practice, and two system-level themes were identified wherein participants described strategies and issues to consider in policy and organizational structure.

### Individual-Level Themes

#### Theme 1: Safety

This theme identifies safety as a fundamental element across all areas of care and service levels. Safety can involve security in the relationship between victim-survivor and practitioner, the physical setting, and addressing immediate safety concerns. Trauma sensitivity and awareness enhance safety by reducing the risk of emotional harm and retraumatization.

##### Safe and Secure Support

Twenty-two studies (see Supplemental Appendix Table B3) discussed the importance of providing safe and secure support, with a focus on safety as a core component of any service provided. Developing safe, supportive relationships with victim-survivors was necessary for safety. One midwife described this in relation to screening procedures: “You want her to feel like she’s being cared for and nurtured so that she can disclose information to you” (Hegarty et al., 2020, p. 73). A “safe space” in terms of physical setting was considered essential, such as using a quiet and secure location (Dawson et al., 2021) and a comfortable, warm, and inviting space (Kahan et al., 2020). Addressing safety concerns involved identifying threats, assessing risk, safety planning, and de-escalation strategies. Safety planning was described as “an iterative process that helps service users identify their current level of safety, identify primary safety concerns, collaborate with [service providers] on ways to increase their level of safety, and in some cases, maintain safety” (Wood et al., 2022, p. 18977). Strategies included identifying and connecting victim-survivors with supports, code words, physical separation from the perpetrator, collecting important items or documents, and organizing emergency accommodation (Willie et al., 2023; Wood et al., 2022). De-escalation was exemplified by hotline advocates using questions such as “Is there a safe place you can go to unwind and get away from the situation?” (Wood et al., 2022, p. 8977).

##### Trauma Awareness for Harm Reduction

Participants across 23 studies (see Supplemental Appendix Table B3) espoused a trauma-informed approach characterized by sensitivity to the impacts of trauma to reduce harm. Service providers recognized that trauma could impact help-seeking, disclosure, and willingness to engage with services. Providers need skill and sensitivity to avoid retraumatizing victim-survivors (Goodman et al., 2016; Hegarty et al., 2020). Incorporating trauma awareness was crucial to promote emotional safety and included awareness of triggering experiences, gentleness during difficult discussions, being clear about boundaries, and ceasing service interactions at appropriate times (Désilets et al., 2020; McGeown et al., 2023). Privacy and confidentiality measures reduced harm, such as information accessible in a private manner and upholding personal space in settings such as shelters (Anderson et al., 2021; Kulkarni et al., 2019). Creative practices were used to ensure privacy and provide safe spaces for women still experiencing abuse to receive support when accessing services for other reasons, these included providing partners or family members with a specific task to complete requiring leaving the room or informing prior to their visit that women were expected to attend this appointment alone (Hegarty et al., 2020). Creative practice was exemplified by an antenatal midwife: “You ask the woman to leave first and then you talk to the partner about. . .how he feels about being a dad then you switch the roles, so they think. . .she’s just going to be asked the same questions and then you ask about the DV.” (Hegarty et al., 2020, p. 73).

#### Theme 2: Survivor-Centered Care

This theme describes the importance of centering victim-survivors’ preferences throughout their service engagement to provide survivor-centered care. This involves information to support choice, matching needs to resources, and providing individualized care to meet the unique priorities and goals of each victim-survivor.

##### Information and Education

Information and education were identified in 18 studies as necessary for allowing victim-survivors to make informed choices that center their needs and priorities. Accurate and accessible information is essential for empowering victim-survivors to make informed choices about matters their supports (Giesbrecht et al., 2023) and prepare victim-survivors for the recovery process (Wood et al., 2021). Providers play a key role in providing advice and educating victim-survivors—and families—about health, DFV, and trauma. Providing information about DFSV was also linked with safety as it supported victim-survivors and family members to identify potential harm and seek help (Wood et al., 2022). Participants used psychoeducation to inform victim-survivors about types of DFSV, trauma responses (e.g., physical reactions) and the impact of different coping mechanisms (e.g., substance use; Willie et al., 2023; Wood et al., 2021). Information reduced the shame and self-blame that victim-survivors experience (Bailey et al., 2020). Service providers noted the potential for harm when victim-survivors receive inaccurate information; thus, services need to remain updated with relevant information (Giesbrecht et al., 2023).

##### Provision of Relevant Resources

The provision of relevant resources was described in 16 studies (see Supplemental Appendix Table B3) as foundational to survivor-centered care. Victim-survivors tend to engage with services to find resources and support options (Wood et al., 2022). Matching victim-survivor support requirements to services and facilitating connections with relevant resources were central to many service providers’ roles (Désilets et al., 2020). Therefore, providers need to know of available resources, with one advocate describing the use of a “mega spreadsheet” (Wood et al., 2022, p. 18973). Participants discussed supporting victim-survivors to identify needs at the outset, making referrals, and establishing contact with a referred service (Armstrong, 2023). Some noted that providing information and referrals is not always enough to engage victim-survivors with support (McConnell & Phelan, 2022). Thus, their role involved guiding and supporting victim-survivors through navigation of various services and supports, particularly as this process can be burdensome for clients. As one campus-based advocate noted: “Just not knowing where things were, or how to do this or that. . . can be exhausting on top of anything else they may be going through” (Wood et al., 2021, p. 33). Service providers also advocated for victim-survivors’ needs and choices when using other services, such as child protection agencies.

##### Catering to Unique, Individual Needs

Service providers recognized the uniqueness of each victim-survivor and their recovery journey, with 20 studies (see Supplemental Appendix Table B3) referring to the need to cater to victim-survivors’ unique, individual needs. Providers across various settings discussed “being survivor-centered [as] being oriented to individual needs” (Wood et al., 2021, p. 32) and “caution[ed] against the one-size-fits-all mentality” (Tarshis et al., 2022, p. 627). To individualize care, service providers assessed victim-survivor needs, goals, and motivating factors at the outset (Williams et al., 2017), with achieving survivor-defined goals fundamental to service interactions (Wood et al., 2021). Supports were identified based on goals through offering suggestions and exploring solutions (Wood et al., 2022). Service providers espoused the need to center women’s perspectives across all aspects of care, such as guiding group interventions (Jolof et al., 2024; Kahan et al., 2020). Participants described tailoring care for different victim-survivors, providing “specialized and adapted” support (Ghidei et al., 2022, p. 10). Participants across studies emphasized the importance of “[doing] their utmost to respond to people as they present” (Bailey et al., 2020, p. 292). Being responsive again involved creativity, as well as flexibility with rules (Jolof et al., 2024), which was particularly salient in DFV shelters where staff described “bending the rules” around holding beds and availability to allow residents to spend time with family or have children stay with them (Goodman et al., 2016).

#### Theme 3: Respectful Emotional Support

Service providers identified respectful emotional support as essential to providing effective TVIC. Emotional support required empathy, compassion, validation, and warmth, with participants providing trauma-focused mental health support and supporting victim-survivors to develop coping and emotion regulation strategies.

##### Empathetic Support

An empathetic approach was described by providers across 24 studies (see Supplemental Appendix Table B3), conveyed through empathic communication (Wood et al., 2022) and helping build relationships with victim-survivors to facilitate service engagement (Agu et al., 2020). Taking the time to understand victim-survivors’ life circumstances allowed providers to be respectful of their preferences and desires by “meet[ing] them where they are at [which] might be different from where we think they should be at” (McConnell & Phelan, 2022, p. 5161). Service providers emphasized letting victim-survivors progress at their own pace, for example, while recalling memories in counseling (Bailey et al., 2020) or allowing time to adjust to shelters through ongoing orientation practices (Kulkarni et al., 2019). A respectful and empathetic approach can be achieved by asking victim-survivors what works for them, incorporating frequent feedback opportunities (Kulkarni et al., 2019), and ensuring victim-survivors feel comfortable to disengage at any time (McGeown et al., 2023), such as by providing accessible, alternative activities during group discussions (Kahan et al., 2020). Additionally, participants identified respectful listening and providing space to be heard as central to empathetic support (Jolof et al., 2024), involving listening carefully and deeply “before you start talking” (McConnell & Phelan, 2022, p. e5161). Providers sought to provide many opportunities for victim-survivors to be heard in a respectful and safe environment, including through group and individual processes, and activities such as having a cup of tea together or going for a walk (Goodman et al., 2016).

##### Compassion and Validation

Compassion and validation were identified across 16 studies (see Supplemental Appendix Table B3) as fundamental to providing effective support. Many participants described their primary role as providing emotional support through “expressing care and interest in [victim-survivors’] lives” (Wood et al., 2022, p. 18971) and at times they could be a victim-survivor’s sole support. A compassionate, caring approach was necessary for successful outcomes: “being authentic, genuine, honest, loving, caring, compassionate, unconditional. . .[are] where [clients] find the success” (Ward-Lasher et al., 2017, p. 209). Compassionate care sought to provide victim-survivors with a “sense of a solid footing” (Goodman et al., 2016, p. 74) and to feel less alone during a difficult recovery journey (Ahrens et al., 2022). Validation and warmth were part of a compassionate approach, achieved using supportive statements such as the following: “This isn’t your fault. I can imagine how hard that would be! It’s important to feel emotionally safe when you get home” (Wood et al., 2022, p. 18970). Validation was used to reduce victim-survivors’ self-blame by conveying reassurance and “[letting] them know someone’s there for them” (Wood et al., 2021). This allowed victim-survivors to build trusting relationships with providers and feel secure enough to be open and promote healing (Désilets et al., 2020).

##### Trauma-Focused Mental Health Support

Service providers in 16 studies (see Supplemental Appendix Table B3) acknowledged the high prevalence of mental health concerns among victim-survivors (Mengo et al., 2020) and recognized the need to address mental health concerns (“mountains the [victim-survivor] must climb before being able to move forward”; Simmons et al., 2017, p. 613), with patience and self-awareness essential (Mengo et al., 2020). Participants supported victim-survivors to process abuse (Ward-Lasher et al., 2017) and change unhelpful patterns of relating to themselves and others (Dawson et al., 2021; Morton et al., 2021). Various counseling and trauma-informed interventions were used, including cognitive behavioral therapy, art therapy, mindfulness-based therapies, parent–child work, and groups. Role-play was used to prepare victim-survivors for difficult interactions with relatives or contact with perpetrators (Goodman et al., 2016). Group work was considered effective as it engendered validation and shared understanding (Bailey et al., 2020), yet was not appropriate for all victim-survivors (van der Hoeven et al., 2023), and safety planning could be used to promote group safety (McGeown et al., 2023).

Providers encouraged supporting the development of coping and emotion regulation strategies that assist victim-survivors to “learn how to relax, self-soothe and manage intense feelings” (Tarshis et al., 2022, p. 628). “Emergency self-care plans” were utilized, which support victim-survivors to identify triggers, recognize symptom escalations, and employ strategies to “self-soothe” (Ward-Lasher et al., 2017, p. 208). Service users emphasized the importance of coping strategies such as mindfulness and grounding techniques to promote “stabilization” (Bailey et al., 2020). The use of self-care was encouraged by incorporating wellbeing-promoting activities and strategies into everyday routines (McConnell & Phelan, 2022). Service providers can support self-care by allowing victim-survivors space to set their worries aside and engage in peaceful, enjoyed activities (van der Hoeven et al., 2023).

#### Theme 4: Non-Judgmental, Reciprocal Client–Practitioner Relationship

This theme describes the importance of developing a non-judgmental, reciprocal client–practitioner relationship, defined by collaboration, mutuality, and recognition of victim-survivors’ lived expertise. This involves a strength-based, non-pathologizing approach that upholds the resilience of victim-survivors and avoids stigmatization.

##### Collaborative Relationship

Service providers across 22 studies (see Supplemental Appendix Table B3) emphasized collaborative relationships between client and practitioner, involving reciprocity, equality, and shared responsibility, wherein “the service user guides the interaction with support. . .and work[ing] collaboratively to identify needs and options that best fit” (Wood et al., 2022, p. 18971). Collaborative relationships were necessary for trauma-informed, survivor-centered care (Kahan et al., 2020; McGeown et al., 2023), and this involved balancing the traditional client–practitioner power dynamic with the service provider as co-learner, “acknowledging the limits of [their] own knowledge” (McConnell & Phelan, 2022, p. e5160) and recognizing lived expertise of victim-survivors. This was implemented through open dialogue between victim-survivors, support network members, and supporting practitioners, which removed “established hierarchies. . .creating space for [victim-survivor] voices” (Dawson et al., 2021, p. 142). Participants encouraged rethinking physical boundaries (e.g., entering the community together) and interpersonal boundaries (e.g., “dropping the professional mask” (Goodman et al., 2016, p. 81)), while some upheld the importance of traditional boundaries for consistent care (Goodman et al., 2016). Clear, honest, and open communication with victim-survivors was considered a necessity. This involved being transparent about service interactions and potential difficulties from the outset, and clear communication about roles, rules, and resources (Kulkarni et al., 2019; McGeown et al., 2023), ensuring victims-survivors knew what to expect (Ward-Lasher et al., 2017).

##### Strengths-Based Approach

A non-judgmental, strength-based approach was described by providers across 18 studies (see Supplemental Appendix Table B3), involving emphasis of victim-survivors’ resilience and internal resources as survivors of DFSV and belief in their ability to grow and change (Kulkarni et al., 2019). Participants described working with survivors to explore and highlight their strengths and courage, drawing upon inbuilt coping resources. A non-judgmental approach was fundamental to strength-based practice, particularly important when working with victim-survivors from diverse cultural groups and abilities (Ghidei et al., 2022; McConnell & Phelan, 2022; van der Hoeven et al., 2023). Service providers espoused avoiding stigmatizing or blaming language, including placing trauma ownership on victim-survivors (e.g., “your assault”) or labeling victim-survivors without their consent (Anderson et al., 2021). Recognizing the pervasive impact of trauma was a key element of the strength-based approach. Participants described seeing challenging behaviors as trauma responses, rather than labeling victim-survivors “difficult” or “resistant” (Armstrong, 2023). Service providers worked with victim-survivors to normalize and reframe their behavior and symptoms (e.g., substance use, unhelpful habits, and difficulty engaging) as “understandable responses to traumatic experiences” (Bailey et al., 2020, p. 292). A non-pathological model was favored over the traditional medical model, with service providers expressing concerns about diagnosing and pathologizing “normal/expected reactions to violence” (Simmons et al., 2017, p. 610) as this could create further harm, as stated by one helping professional: “Victims in crisis mode don’t need to be “labeled” as having mental health issues as well. The abuser has probably told them that over and over” (Simmons et al., 2017, p. 610).

#### Theme 5: Supporting Victim-Survivors to Thrive

This theme extends recovery beyond safety and survival to victim-survivors “thriving” and enjoying life in the aftermath of DFSV, involving a process of rebuilding and finding meaning through overcoming the damage to a victim-survivor’s agency and sense of self, promoting social connections, community reintegration, and exploring new skills and interests.

##### Supporting Healthy Social Connections

Promoting healthy social connection was identified by service providers across 12 studies (see Supplemental Appendix Table B3) as a key goal in supporting recovery. Participants encouraged victim-survivors to reconnect with pre-existing supports and communities or establish new connections. This involved navigating potential challenges, with coaching and role-playing used to “help survivors anticipate potential bumps in communication, manage expectations, and deal with rejection” (Goodman et al., 2016, p. 74). Support plans were developed to encourage engagement in community or cultural activities and provide opportunities to form friendships, including with other victim-survivors (Ahrens et al., 2022; McConnell & Phelan, 2022). Peer support connections provided victim-survivors a sense of hope and altruism from helping others (Kahan et al., 2020; McGeown et al., 2023) and were a source of validation, as victim-survivors “find recognition and acknowledgment in each other” (van der Hoeven et al., 2023, p. 520). Practitioners facilitating groupwork acknowledged the need to address safe communication and potential risks when victim-survivors connect with other group members (Goodman et al., 2016). Social and community connections were identified as particularly important for victim-survivors from migrant communities, university students, LGBTQIA+, and those with intellectual disability due to the risk of isolation.

Participants recognized that abuse can damage victim-survivors’ understandings of healthy relationships (Armstrong, 2023); as such, practitioners worked with victim-survivors to develop their ability to form healthy relationships, by assessing the safety of relationships and developing skills around boundary setting, communication, and assertiveness in relationships (McConnell & Phelan, 2022; Wood et al., 2021). One shelter practitioner described “helping survivors relearn to communicate with others in a way that did not produce shame on the survivor’s part” (Goodman et al., 2016, p. 71). An aim is to develop new templates for safe social connections, with an example being a “Web of Safety” drawn with victim-survivors, where solid lines depict safe relationships and dotted lines indicate possible connections with the perpetrator (Goodman et al., 2016).

##### Promoting Agency and Sense of Self

Service providers across 21 studies (see Supplemental Appendix Table B3) discussed the importance of promoting victim-survivor agency and identity, affirming their sense of self and incorporating the principles of choice, control, and empowerment. Participants acknowledged that victim-survivors experience a profound loss of autonomy through DFSV, and seeking help can recreate this sense of powerlessness (Dawson et al., 2021; Hegarty et al., 2020). Nurturing the independence and autonomy of victim-survivors was thus a primary goal for many service providers, with empowerment a central component. A campus-based advocate described the purpose of her work as the following: “Definitely being survivor-centered and trauma-informed and so empowerment, choice, and control are really important for the student leading what their advocacy appointment looks like or meeting their needs” (Wood et al., 2021, p. 32). Supporting choice was considered essential to a positive outcome, with success defined as the victim-survivor “feeling comfortable and confident with her decision and her choice” (Agu et al., 2020, p. 447). Some participants were conscious of victim-survivors becoming dependent on them and managed this through support and preparation (Goodman et al., 2016). Service providers acknowledged that victim-survivors experience difficulties with “self-affirmation, self-esteem and knowing oneself” (Désilets et al., 2020, p. 180). They sought to affirm victim-survivors’ sense of self through “building internal foundations” (Goodman et al., 2016, p. 70), helping them “transition from a world schema based on their sense of self as ‘mad or bad’ to one of positive self-identity” (Bailey et al., 2020, p. 294). McConnell and Phelan (2022) described supporting victim-survivors to “reintegrate a narrative that affirms their self-worth and seeds hope” (p. e5163) through helping them better understand their selves and histories, and to identify, or reidentify, their needs, emotions, interests, and goals.

##### Rebuilding and Improving Victim-Survivors’ Lives

Victim-survivors engage in a process of rebuilding their lives when recovering, and service providers across eight studies (see Supplemental Appendix Table B3) described supporting victim-survivors to both reconstruct and improve their lives. Participants observed how interventions such as art therapy, counseling, and sharing stories allowed for growth and healing (Morton et al., 2021). Service providers aimed to support victim-survivors to find meaning, hope, and a sense of enjoyment after DFSV. This was achieved through encouraging victim-survivors to explore interests, and assisting with brainstorming, finding resources, overcoming barriers, and transporting or accompanying them (Goodman et al., 2016; McConnell & Phelan, 2022; van der Hoeven et al., 2023). Advocates connected victim-survivors with “religious settings, help[ed] them find scholarships to attend school, and help[ed] them access specific clubs, groups, or classes” (Goodman et al., 2016, p. 75), such as communal meals, libraries, arts and crafts, Karate classes, dancing groups, and free concerts (Bailey et al., 2020). Community activities were meaningful opportunities for victim-survivor healing. Practitioners also encouraged skill-building, with a particular focus on “life-skills” (Wood et al., 2021).

#### Theme 6: Cultural Safety and Inclusivity

Cultural safety and inclusivity were considered fundamental across all areas of service engagement. Findings demonstrated how incorporating these values into provider practices were vital to engaging in violence-informed practice and preventing harm to the array of victim-survivors that engage with services, from diverse cultural, immigration status, religious, gender, sexual orientation, ability, health status, and socioeconomic groups.

##### Intersectionality

Intersectionality, or the interaction between types of discrimination and oppression, was discussed by participants across 20 studies (see Supplemental Appendix Table B3) as a core component of TVIC, often in relation to working with migrant, Indigenous, or gender-diverse victim-survivors. Participants had witnessed discrimination against victim-survivors from marginalized groups (Giesbrecht et al., 2023) and acknowledged that fear of judgment and rejection were barriers to help-seeking. Service providers thus advocated “be[ing] sensitive to the multiple realities that women face” (Désilets et al., 2020, p. 180) using an approach that acknowledges the complex social contexts in which violence occurs (Wood et al., 2021). Participants described using a “holistic assessment” to examine all types of abuse (Hegarty et al., 2020). Compassion, patience, and a trusting environment were required to practice through an intersectional lens (Désilets et al., 2020). Participants also addressed intergenerational trauma (Morton et al., 2021), and engaged in conversations with victim-survivors about experiences of discrimination, racism, and oppression (Tarshis et al., 2022). Participants recognized DFSV as embedded in patriarchal beliefs, arguing that trauma work needs to be grounded in “the culture of women-hating” as this is “the underlying foundational piece” (Armstrong, 2023).

##### Inclusive Practice

Service providers across 20 studies (see Supplemental Appendix Table B3) described inclusive practices to increase accessibility and effectively support victim-survivors with a diverse range of circumstances and support requirements. Inclusive practices included providing resource information tailored to different groups (e.g., LGBTQIA+-specific hotlines or community spaces; Anderson et al., 2021), allowing flexibility in when and how victim-survivors access support (Mengo et al., 2020), and collaborating with inclusive services for advice (Wood et al., 2021). Use of language that is gender neutral and accessible for victim-survivors with lower literacy skills, age, education and experience was necessary (Anderson et al., 2021; Kahan et al., 2020). When working with people with intellectual disability, participants emphasized internal capabilities, incorporating choice, control, and solidarity and ensuring referred services were respectful and matched to their ability level (McConnell & Phelan, 2022). For victim-survivors in rural settings, confidentiality, privacy, and managing boundaries were key considerations (Hegarty et al., 2020) Welcoming and non-judgmental spaces were important for engaging victim-survivors experiencing homelessness (Kahan et al., 2020) and parenting support was highlighted (Jolof et al., 2024) by “working with survivors on building parenting skills in a respectful way. . . providing parenting respite even if just for a few minutes” (Kulkarni et al., 2019, p. 153).

##### Cultural Safety

Practices promoting cultural safety were highlighted by providers across 14 studies (see Supplemental Appendix Table B3) as essential to TVIC. Service providers emphasized “respect[ing] people’s different cultures, faiths and traditions” (Ghidei et al., 2022, p. 10) and the need for cultural humility, awareness, a non-judgmental approach, and critical self-reflection of biases (Agu et al., 2020; Wekerle et al., 2022) as well as asking and not assuming victim-survivors’ preferences (Giesbrecht et al., 2023). Cultural understanding and trauma impacted help-seeking as victim-survivors can be unaware DFV is occurring (Anderson et al., 2021) or feel unsafe seeking help (Ghidei et al., 2022). Findings indicated that service providers need to be sensitive to the impacts of colonialism and cultural trauma, particularly when working with Indigenous victim-survivors (Wekerle et al., 2022). Indigenous providers described the importance of “respecting the role of the community” and “connection to family/kinship/clanship as key facets of wellbeing” for Indigenous communities (Wekerle et al., 2022, p. 10). Western practices were not always suitable, and providers emphasized the need to incorporate norms and values of other cultures into their practice, including a greater emphasis on relationship-building and indirect approaches to care as opposed to more direct styles (Ahrens et al., 2022). Additional practices involved supporting victim-survivors to participate in cultural activities (e.g., shopping at a culturally specific grocery store (Goodman et al., 2016), providing opportunities to say “no” when needs were not being met, and providing language-specific resources and culturally relevant materials (Giesbrecht et al., 2023; Kulkarni et al., 2019).

### System-Level Themes

#### Theme 7: Provision of Effective and Accessible Services

This theme describes the principles and methods that can be applied at the system level to increase effectiveness and accessibility of services, including inclusivity, flexibility, and reduced complexity. Continuity of care was identified as reducing harm, and service improvement strategies included matters relating to staff and screening practices.

##### Flexible Services with Reduced Complexity

Service providers across 11 studies (see Supplemental Appendix Table B3) recognized that providing flexible services with reduced complexity can increase accessibility and overcome barriers to support and engagement. Flexibility in when/how often service engagement occurred increased supportiveness (Hegarty et al., 2020), with longer term services considered more beneficial (McGeown et al., 2023). Group practitioners at shelters overcame barriers to engagement by scheduling evening groups and phone availability (Kahan et al., 2020). Participants encouraged reduction of restrictive rules at women’s shelters to increase accessibility (Simmons et al., 2017), such as removing curfews, reconsidering absolute sobriety rules, and stay extension flexibility (Kulkarni et al., 2019). The importance of timely access to services was also discussed, supported using chat, app-based, and hotline services (Anderson et al., 2021; Wood et al., 2021). Simplicity and directness were means of reducing complexity and increasing accessibility, such as providing information in simplified language (Anderson et al., 2021), reducing paperwork (Ahrens et al., 2022), and direct, clear screening methods (Hegarty et al., 2020).

##### Continuity of Care

The need for continuity of care was highlighted by service providers across nine studies (see Supplemental Appendix Table B3). Without it, victim-survivors were required to retell their stories numerous times and “recalling traumatic experiences can be re-victimizing” (Williams et al., 2017, p. 826), thus, services reducing this requirement were advocated (Wood et al., 2022). Service providers advocated for having one point of access (Wood et al., 2021) and promoted continued engagement through supportive and encouraging language (Wood et al., 2022). Furthermore, interacting with the same service provider each time was considered beneficial in promoting relationship-building, safety, and help-seeking (Ahrens et al., 2022; Jolof et al., 2024), increased consistency, and a thorough understanding of victim-survivor priorities (Goodman et al., 2016, p. 82). Not terminating services prematurely, leaving victim-survivors without contact or needed support was also considered important.

##### Inclusive Services

Service providers across 12 studies described system-level practices that promote inclusivity to meet the needs of different victim-survivors (see Supplemental Appendix Table B3). Concerns were expressed around services adequately meeting the priorities of certain groups, such as cultural minority and LBTQGIA+ populations. Services needed more funding to hire additional staff, provide longer-term support, and increase availability of counseling, support for children, and access to legal assistance (Morton et al., 2021). “Second-stage shelters” providing housing for victim-survivors for 1 to 2 years and the use of outreach workers supporting victim-survivors after leaving shelters were recommended (Giesbrecht et al., 2023). Providers acknowledged the importance of integrating services with “more approachable and culturally acceptable” community settings, that is, locating them at the same location as churches or community centers (Ahrens et al., 2022, p. 212) and training peer leaders to serve as community supports. The need for more cultural competence training was expressed, including more localized information and potentially retraumatizing practices (Wekerle et al., 2022). Offering services in different languages was essential, either using staff from different language backgrounds or interpreters (Anderson et al., 2021; Ghidei et al., 2022). Some concerns around interpreter confidentiality were noted; participants recommended using remote, telephone interpreting services or interpreters not living locally.

##### Service Improvement Strategies

Additional service improvement strategies were described across 26 studies (see Supplemental Appendix Table B3). Many strategies are related to staff education, training, and support. Service providers acknowledged feeling underprepared, and more training was encouraged (Jolof et al., 2024; Mengo et al., 2020; Willie et al., 2023), particularly around DFV, conflict/de-escalation strategies, culture and intersectionality, working with children, trauma, mental health, and disclosure response. Supporting staff and facilitating their self-care were considered necessary, including providing feedback and guidance, stress relief activities (Agu et al., 2020), time off (Ward-Lasher et al., 2017), peer support, back-up staff during crises, “restorative physical spaces” and wellness opportunities, and supervision (Kulkarni et al., 2019; McGeown et al., 2023). Services were encouraged to provide the “places, space, and opportunity for staff to heal as much as survivors” (Kulkarni et al., 2019, p. 150). The importance of a culturally safe, trauma-and-violence-informed, survivor-centered, and anti-oppressive organizational culture at all service levels was espoused. Clear, uniform approaches to screening were encouraged (Hegarty et al., 2020).

#### Theme 8: Providing Holistic Services

This theme describes the importance of supporting victim-survivor recovery in its entirety—through provision of holistic services, incorporating a range of services within organizations and collaboration between services. This included expanding the reach of services to the community level by working to promote community awareness and prevention.

##### Comprehensively Meeting Needs

Participants across 22 studies described providing holistic services to comprehensively meet victim-survivor needs, acknowledging that victim-survivors possess a range of support requirements across an array of services. These included “shelters, hotlines, legal services, counseling, education, employment assistance, childcare assistance, housing assistance, financial assistance, and immigration assistance” (Ahrens et al., 2022, p. 212). A “no wrong door” approach was favored as some victim-survivors “have been passed around from one agency to another to another, sometimes for years. . . not getting their problems addressed” (Armstrong, 2023). Providers recognized that challenges with housing, parenting, employment, and immigration serve as obstacles to victim-survivor recovery. Therefore, participants espoused holistic services addressing victim-survivor needs in their entirety and considering those most pressing, in particular, the basic needs of financial, employment, housing, and parenting considerations (Willie et al., 2023). TIC was identified as involving holistic support, as in the following: “Part of the wonderful thing about [TIC]. . . is that whatever issues they come to the table with, you’re going to. . . address them” (Mengo et al., 2020, p. 186). Collaboration between services and partnerships with other services, agencies, and community groups facilitated holistic support (Agu et al., 2020; Armstrong, 2023); service providers also illustrated how holistic services “respond to the needs of the family” as a whole (Tarshis et al., 2022, p. 628) by supporting and communicating with family members.

##### Community Engagement and Prevention

Community engagement and prevention were discussed by participants across eight studies. Service providers recognized that DFSV is a challenge for the entire community, requiring community-level advocacy (Wekerle et al., 2022). Participants described how services were used as “community education tools” by raising awareness and providing the community information about DFSV through health and campus-based services and support hotlines (Willie et al., 2023; Wood et al., 2021; Miller et al., 2017). Raising community awareness was seen as preventive. Additionally, it was important that practitioners understood the communities in which they work, as described by one HIV/DFV service provider: “We need to be sensible, sensitive, and competent in terms of knowing the issue and the community that you’re providing that service to” (Williams et al., 2017, p. 826). Service providers recognized that their services could create a “ripple effect” (Miller et al., 2017), empowering women to help others in the community,

## Discussion

This meta-synthesis integrated the findings from qualitative studies using a systematic search method aiming to determine what constitutes effective, TVIC for victim-survivors of DFSV from the perspectives of service providers. The extant literature demonstrates that service providers espoused an approach that is holistic, recovery-oriented, trauma-and-violence-informed, strength-based, respectful, and survivor-centered, incorporating considerations of intersectionality, empathy, safety, mutuality, and meeting the unique priorities and support requirements of all victim-survivors. Thematic synthesis derived eight themes relating to elements of effective TVIC, describing practices and approaches at the individual- and system-level. The critical findings and implications of this review are displayed in Tables 1 and 2, respectively. Findings provide a healing-oriented framework outlining the principles, practices, and approaches required to support victim-survivor recovery and wellbeing, characterized by equity and empowerment. These principles can be incorporated in future studies to develop indicators for evaluating DFSV service delivery.

**Table 1.** Critical Findings of This Study.

Eight Identified Themes Derived from the Studies	
Individual-level themes	
1.	Safety: Safety was identified across most studies and recognized to be fundamental to any support provided, involving safety in the client–practitioner relationship, the physical setting, and incorporating trauma-informed practices to reduce harm.
2.	Survivor-centered care: Service providers across many studies described the importance of centering victim-survivors’ needs, preferences, and perspectives throughout service engagement and individualizing care to meet unique needs.
3.	Respectful emotional support: A recurring theme across studies was the use of a respectful and empathetic approach to care, incorporating compassion, validation, and warmth, and addressing mental health concerns in a manner that is sensitive to trauma and promotes healthy regulation strategies.
4.	Non-judgmental, reciprocal client-practitioner relationship: The client-practitioner relationship was described across many studies as one that should be reciprocal, equal, non-judgmental, and strength-based, involving collaboration, mutuality, and upholding the resilience and lived expertise of victim-survivors.
5.	Supporting victim-survivors to thrive: Service providers recognized recovery as a process extending far beyond safety and survival, describing supporting victim-survivors to thrive through promoting social connection, reengagement with the community, empowerment and agency, affirming identity, and finding meaning, hope, and enjoyment.
6.	Cultural safety and inclusivity: The principles of cultural safety and inclusivity are foundational to a trauma-and-violence-informed approach, described across many studies as incorporating cultural competence, awareness, and understanding, inclusive practices to increase accessibility, and recognizing intersectionality.
System-level themes	
7.	Provision of effective and accessible services: Practices were described at the system level to improve services and provide more effective and accessible support, including increased flexibility, reduced complexity, promoting continuity of care, inclusivity, and supporting staff.
8.	Providing holistic services: The importance of a holistic approach to support was recognized across many studies in order to effectively meet the needs of victim-survivors and promote recovery and wellbeing, this included engaging with the community to promote engagement and prevention.

**Table 2.** Implications of the Review for Practice, Policy, and Research.

Type	Implications
Practice	• The trauma- and violence-informed approach to supporting victim-survivors of DFSV entails practitioners’ centering safety and harm reduction, providing survivor-centered care through matching needs to resources and catering to unique, individual needs, providing compassionate and empathetic emotional support, developing collaborative, non-judgmental relationships with victim-survivors, emphasizing their strengths, supporting victim-survivors to rebuild their lives, promoting agency, and always engaging in culturally safe, inclusive practices. • Practitioners provide a range of supports to facilitate recovery and healing, including support with mental health and wellbeing, as well as emotional, social, family, practical, and material support.
Policy	• Policymakers need to support the consistent application of trauma-and-violence-informed approaches across services to promote recovery and healing and minimize harm. • Victim-survivors’ basic needs must be addressed through policies that promote affordable housing, affordable and quality childcare, employment opportunities, financial support, with additional attention to victim-survivors from marginalized groups.
Research	• Evaluation into DFSV service systems utilizing the TVIC framework is necessary to determine its efficacy and improve outcomes for victim-survivors. • Further research is needed into practices that support children affected by DFSV and improving services for children.

*Note.* DFSV = domestic, family, and sexual violence; TVIC = trauma-and-violence-informed care.

The results of this review align with and extend existing frameworks for implementing TIC and TVIC (SAMHSA, 2014; Wathen & Mantler, 2022). The findings reinforce and operationalize SAMHSA’s (2014) TIC principles within domestic violence-specific contexts, highlighting the importance of safety, empowerment, and mutuality, yet expanding upon these to incorporate important considerations of power, structural violence, intersectionality, and systemic and equity-oriented practices. In exploring TVIC, the synthesized themes strongly align with and expand upon Wathen and Varcoe’s (2023) principles of TVIC including considerations around choice, collaboration, and connection, systemic and structural violence, different types of safety, and strength-based approaches to recovery. The current findings extend TVIC—and previous reviews of TIC (Wilson et al., 2015)—by highlighting the need for multidimensional conceptualizations of recovery, the fundamental importance of client–practitioner relational dynamics, rebalancing power through reflexivity, and including both individual- and system-level practices to operationalize the “violence-informed” approach and further incorporate considerations of intersectionality and cultural safety across service settings that work with diverse populations.

The findings emphasized the relational nature of TVIC, demonstrating that it not only involves employing specific practices but embodying an approach characterized by humility, empathy, and authenticity. These considerations are particularly important in a DFSV context to avoid repeating the abusive dynamics that victim-survivors have previously experienced (Trevillion et al., 2016). While DFSV and trauma-specific knowledge is required, the strong emphasis on relational dynamics in the current themes suggests that communication and relational skills are essential in mitigating retraumatization and promoting positive outcomes, including emotional attunement, respectful listening, and centering lived expertise. Non-judgmental, strength-based practice involves upholding the resilience and internal resources of victim-survivors, and providers must be willing to relinquish the power implicit in a traditional client-practitioner relationship, acknowledge their limits, and engage in a process of co-learning and collaboration with victim-survivors to provide empowerment-based support. This is supported by previous research indicating greater empowerment and control for victim-survivors when accessing services is related to improved safety, mental health, and increased help-seeking (Cattaneo & Goodman (2015). Through empathetic, non-judgmental approaches, centering victim-survivors’ preferences and safety, and promoting empowerment and agency, TVIC advocates a collaborative approach that places victim-survivors as experts in their own recovery rather than passive recipients of care.

The overall approaches and principles identified reflect those considered important for working with victim-survivors throughout the service system to mitigate retraumatization, support recovery, and increase service engagement. However, the TVIC approach can be applied differently across settings. For example, respectful listening can include reassurance and warmth in a therapeutic environment, validating statements or extra time to listen over hotlines, ensuring safety to share in group settings, and accompanying victim-survivors for a walk or a cup of tea in advocacy and shelter settings.

The importance of a culturally relevant approach across all areas of service engagement was emphasized by service providers across studies, involving respecting culture and tradition, adapting support in line with preferences and norms, language accessibility, integrating services with community settings, and sensitivity to the impacts of colonization and historical trauma. Cultural safety and inclusivity are particularly important due to the experiences of discrimination and stigmatization that victim-survivors from marginalized groups experience when accessing services (Kennedy et al., 2023; Murvartian et al., 2023; Taylor, 2020) and the cultural barriers to help-seeking (Green et al., 2024). Providers outlined an approach to service provision that both recognizes and addresses the complex realities that victim-survivors experience, highlighting the need to listen to diverse voices and adapt support to the unique contexts, identities, and intersecting oppressions experienced by each victim-survivor. The findings suggest that cultural safety is not a static competency but involves an ongoing process of critical self-reflection and revision of existing practices and systems that emphasize Western notions of wellbeing and service interaction, highlighting the need for co-design of services with members of culturally diverse groups. This is particularly relevant to the high-income, Western, multicultural countries from which the results came.

The findings conceptualized recovery as a multidimensional concept, extending beyond safety and physical concerns, aligning with previous literature recognizing recovery as a process of not only healing the damaging effects of DFSV but moving toward optimal functioning, achieving satisfaction and meaning across life domains (Flasch et al., 2017). Service providers promoted victim-survivor healing far beyond safety and survival, supporting them to rebuild their lives, finding meaning and hope through affirming agency and identity and supporting healthy social connections and community. The importance of promoting social connection is not surprising given that social support is considered one of the most important factors in victim-survivor recovery (Blasco-Ros et al., 2010). The service provider role outlined in the results was comprehensive and involved providing emotional, practical, material, and social support to promote recovery and healing across all domains, outlining the need for holistic supports. The multifaceted nature of recovery means that services should consider victim-survivors’ own definitions of healing and empowerment when designing and considering service outcomes. Furthermore, the complex, long-term, and relational nature of recovery should be reflected in service timeframes and availability.

The current findings emphasize the notion that TVIC must be implemented at all levels of an organization and system to effectively respond to DFSV. Service providers identified system-level strategies to increase accessibility and availability of services, ensure continued care, comprehensively support victim-survivors, and improve organizational culture. Service systems are hindered by inadequate funding and resource constraints (Emsley et al., 2022), indicating a need for greater public awareness and recognition at the policy level of the cruciality of DFSV services, to increase funding, staffing, and training opportunities. The need for holistic support and recognition of the overlapping challenges that victim-survivors face identifies that economic, housing, parenting, and employment support are inseparable from recovery. This aligns with research into the social determinants of health, which additionally notes these factors are rooted in structural stratifiers, such as gender, race, and social class (WHO, 2010). Therefore, to effectively promote recovery and healing, integrated service systems are required, and it is critical that policymakers create legislation addressing social, structural, and income inequality that promote financial support, affordable housing, quality childcare, and employment opportunities.

### Strengths and Limitations

A strength of this meta-synthesis is the use of a systematic, rigorous search strategy to capture service providers’ perspectives from a range of services and professions. The participants specialized in providing services to a broad, diverse range of victim-survivor populations, including newly migrated or culturally diverse victim-survivors, Indigenous populations, women with intellectual disability, the LBGTQIA+ community, and those experiencing homelessness. The findings are thus generalizable to the needs of a large proportion of victim-survivors and capture unique information about working with those from diverse and marginalized groups who would be most at risk of experiencing stigmatization and challenges with services. As a result, this review more extensively addressed inclusivity, diversity, and cultural safety than previous research. Furthermore, the findings comprehensively incorporated principles, approaches, and specific practices to provide effective care at both the individual-level and system-level.

This meta-synthesis is not without its limitations. The risk of bias through inclusion of low-quality studies needs to be considered when interpreting results. However, this allowed for the capturing of underrepresented voices and greater breadth of information. Despite the diversity of victim-survivor populations, the 26 included studies were conducted in only 7 high-income, Western countries. Therefore, findings may not be generalizable to middle-income, low-income, or non-Western countries. Given the high rates of DFSV across the globe and low levels of help-seeking (Stiller et al., 2025; WHO, 2021), further research into effective care for victim-survivors of DFSV in non-Western countries or different economic circumstances are required. While the current review aimed to synthesize optimal approaches, principles, and practices across the DFSV sector, it is unclear to what extent these practices are consistently implemented, the variation in approaches between services, and whether their implementation has benefited victim-survivors. Victim-survivors’ perspectives should thus be simultaneously considered along with providers’ views. Further research synthesizing findings related to child and adolescent victim-survivors is also necessary and recommended.

## Conclusion

Victim-survivors of DFSV have unique priorities and support requirements to consider when providing services to support their recovery from trauma, and services need to be better equipped at providing effective care. This meta-synthesis comprehensively reviewed the literature about service providers’ perspectives on providing care in the DFSV sector. This is the first qualitative meta-synthesis to examine what constitutes effective TVIC for victim-survivors of DFSV across services. The themes and subthemes outline a framework describing approaches, principles, and practices that can be incorporated into service provision at both the system and individual level. The findings of this meta-synthesis can inform practitioners and services on how to provide better support for victim-survivors of DFSV with the intention of improving service outcomes and promoting recovery and healing in the aftermath of DFSV.

## Supplemental Material

sj-docx-1-tva-10.1177_15248380251383933 – Supplemental material for Trauma-and-Violence-Informed Care for Victim-Survivors of Domestic, Family and Sexual Violence: A Qualitative Meta-Synthesis of Service Providers’ Perspectives Supplemental material, sj-docx-1-tva-10.1177_15248380251383933 for Trauma-and-Violence-Informed Care for Victim-Survivors of Domestic, Family and Sexual Violence: A Qualitative Meta-Synthesis of Service Providers’ Perspectives by Mia Davies, Lata Satyen and John W. Toumbourou in Trauma, Violence, & Abuse

sj-docx-2-tva-10.1177_15248380251383933 – Supplemental material for Trauma-and-Violence-Informed Care for Victim-Survivors of Domestic, Family and Sexual Violence: A Qualitative Meta-Synthesis of Service Providers’ Perspectives Supplemental material, sj-docx-2-tva-10.1177_15248380251383933 for Trauma-and-Violence-Informed Care for Victim-Survivors of Domestic, Family and Sexual Violence: A Qualitative Meta-Synthesis of Service Providers’ Perspectives by Mia Davies, Lata Satyen and John W. Toumbourou in Trauma, Violence, & Abuse
