# Supplementary material for: Trauma-and-Violence-Informed Care for Victim-Survivors of Domestic, Family and Sexual Violence: A Qualitative Meta-Synthesis of Service Providers’ Perspectives
Source: Trauma Violence Abuse. 2025 Nov 4;27(5):2615–32. doi: 10.1177/15248380251383933 (PMC13736284; doi:10.1177/15248380251383933)
Supplement: Supplementary file 1 [file sj-docx-1-tva-10.1177_15248380251383933.docx]

# Appendix A

Search Strategy

Search terms, displayed in Table 1, were applied to the title and abstract of papers. Subject headings, as displayed in Table 2, were used for all databases and an English language limiter was applied across databases. SCOPUS was additionally used for snowball citation searching.

**Table 1**

*Search Terms*

| Search number | Search syntax |
| --- | --- |
| 1 | “trauma-informed” N3 (care OR support* OR approach* OR practice* OR strateg* OR treatment OR intervention* OR recover* OR therapy OR model OR framework* OR guideline* OR principle* OR style OR service* OR program OR provision OR implementation OR agenc*)  OR ( “trauma aware” N3 (care OR support* OR approach* OR practice* OR strateg* OR treatment OR intervention* OR recover* OR therapy OR model OR framework* OR guideline* OR principle* OR style OR service* OR program OR provision OR implementation OR agenc*) )  OR ( “trauma responsive” N3 (care OR support* OR approach* OR practice* OR strateg* OR treatment OR intervention* OR recover* OR therapy OR model OR framework* OR guideline* OR principle* OR style OR service* OR program OR provision OR implementation OR agenc*) )  OR ( “trauma-focused” N3 (care OR support* OR approach* OR practice* OR strateg* OR treatment OR intervention* OR recover* OR therapy OR model OR framework* OR guideline* OR principle* OR style OR service* OR program OR provision OR implementation OR agenc*) )  OR ( “wom?n-centred” N3 (care OR support* OR approach* OR practice* OR strateg* OR treatment OR intervention* OR recover* OR therapy OR model OR framework* OR guideline* OR principle* OR style OR service* OR program OR provision OR implementation OR agenc*) )  OR ( “trauma sensitive” N3 (care OR support* OR approach* OR practice* OR strateg* OR treatment OR intervention* OR recover* OR therapy OR model OR framework* OR guideline* OR principle* OR style OR service* OR program OR provision OR implementation OR agenc*) )  OR ( retraumati?* N3 (care OR support* OR approach* OR practice* OR strateg* OR treatment OR intervention* OR recover* OR therapy OR model OR framework* OR guideline* OR principle* OR style OR service* OR program OR provision OR implementation OR agenc*) )  OR ( “victim-cent?ed” N3 (care OR support* OR approach* OR practice* OR strateg* OR treatment OR intervention* OR recover* OR therapy OR model OR framework* OR guideline* OR principle* OR style OR service* OR program OR provision OR implementation OR agenc*) )  OR ( “recovery-oriented” N3 (care OR support* OR approach* OR practice* OR strateg* OR treatment OR intervention* OR recover* OR therapy OR model OR framework* OR guideline* OR principle* OR style OR service* OR program OR provision OR implementation OR agenc*) )  OR ( “violence-informed” N3 (care OR support* OR approach* OR practice* OR strateg* OR treatment OR intervention* OR recover* OR therapy OR model OR framework* OR guideline* OR principle* OR style OR service* OR program OR provision OR implementation OR agenc*) )  OR ( “trauma-specific” N3 (care OR support* OR approach* OR practice* OR strateg* OR treatment OR intervention* OR recover* OR therapy OR model OR framework* OR guideline* OR principle* OR style OR service* OR program OR provision OR implementation OR agenc*) )  OR ( “trauma-and-violence-informed” N3 (care OR support* OR approach* OR practice* OR strateg* OR treatment OR intervention* OR recover* OR therapy OR model OR framework* OR guideline* OR principle* OR style OR service* OR program OR provision OR implementation OR agenc*))  OR ( “survivor-cent?ed” N3 (care OR support* OR approach* OR practice* OR strateg* OR treatment OR intervention* OR recover* OR therapy OR model OR framework* OR guideline* OR principle* OR style OR service* OR program OR provision OR implementation OR agenc*))  OR (“attention to trauma” N3 (care OR support* OR approach* OR practice* OR strateg* OR treatment OR intervention* OR recover* OR therapy OR model OR framework* OR guideline* OR principle* OR style OR service* OR program OR provision OR implementation OR agenc*) ) |
| 2 | practitioner* OR clinician* OR advocate* OR worker* OR staff OR volunteer* OR specialist OR “social work*” OR psychologist* OR doctor* OR nurs* OR “medical professional*” OR “healthcare worker*” OR “healthcare professional*” OR “social support” OR “legal support” OR “housing support” OR solicitor* OR lawyer* OR barrister* OR “legal professional*” OR “trauma service*” OR organi?ations OR organi?ation OR “service provider*” OR professional* OR shelter* OR housing OR “homelessness service*” OR administrator* OR “case manager*” OR coordinator* OR personnel OR consultant* OR physician* OR police* OR “allied health” OR expert* OR employee* OR stakeholder* OR policy OR staff OR researcher* OR observer* |
| 3 | “partner violence” OR “partner abuse” OR “dating violence” OR “couple violence” OR “relationship violence” OR “domestic violence” OR “domestic abuse” OR “family violence” OR “marital violence” OR “spous* violence” OR “spous* abuse” OR “battered wom?n” OR “wife battering” OR “wife beating” OR “sexual violence” OR “gender-based violence” OR “gendered violence” OR “violence against women” OR “sexual assault” |
| 4 | perspective* OR opinion* OR attitude* OR view* OR experienc* OR preference* OR desire* OR barrier* OR seek* OR influenc* OR choice* OR benefit* OR positive OR outcome* OR consequence* OR effective OR hope* OR preference* OR “help-seeking” OR desire* OR choice* OR principle* OR approach* OR application* OR need* OR impact* OR challenge* OR opportunit* OR way* OR success* OR empower* OR lens |
| 5 | 1 AND 2 AND 3 AND 4 |

**Table 2**

*Subject Headings and Limiters*

| Database | Subject headings |
| --- | --- |
| MEDLine Complete | Intimate Partner Violence, Domestic Abuse, Spouse Abuse, Battered Women, Nurse Practitioners, Nurses, Occupational Groups, Volunteers, Social Workers, Lawyers, Police, Health Personnel, Case Managers, Psychotherapists, Physicians, Nursing Staff, Hospital Personnel, Medical Staff, Allied Health Personnel, Counselors |
| CINAHL Complete | Intimate Partner Violence, Domestic Violence, Dating Violence, Battered Women, Gender-Based Violence, Health Personnel, Allied Health Personnel, Case Managers, Community Health Workers, Medical Staff, Mental Health Personnel, Psychologists, Psychotherapists, Physicians, Volunteer Workers, Rural Health Personnel |
| PsychINFO | Intimate Partner Violence, Domestic Violence, Dating Violence, Battered Females, Gender Violence, Trauma-Informed Care, Trauma Treatment, Clinicians, Clinician Practice, Mental Health Services, Community Mental Health Services, Social Services, Community Services, Shelters |
| SocINDEX | Intimate Partner Violence, Family Violence, Marital Violence, Rape in Marriage, Wife Abuse, Dating Violence, Relationship Abuse, Domestic Violence, Women’s Shelters, Social Workers, Human Services Personnel, Social Services |
| Global Health | Spouse Abuse, Trauma, Workers, Careproviders, Experts, Health Care Workers, Nurses, Personnel, Physicians, Social Workers, Occupations |
