# Supplementary material for: Trauma-and-Violence-Informed Care for Victim-Survivors of Domestic, Family and Sexual Violence: A Qualitative Meta-Synthesis of Service Providers’ Perspectives
Source: Trauma Violence Abuse. 2025 Nov 4;27(5):2615–32. doi: 10.1177/15248380251383933 (PMC13736284; doi:10.1177/15248380251383933)
Supplement: Supplementary file 2 [file sj-docx-2-tva-10.1177_15248380251383933.docx]

**Appendix B**

**Table B1**

*Methodological Characteristics of Included Studies*

| Authors (year) | Participants (*N*) | Gender identification of participants | Cultural background/ethnicity of participants | Experience of participants | Country | Study design | Analysis |
| --- | --- | --- | --- | --- | --- | --- | --- |
| Jolof et al. (2024) | Service providers at centers for forced migrants (32) | 28 women (87.5%) | NR (“a range”) | Median 1.5 years (range 0–9 years) | Sweden | Focus group | Systematic text condensation |
| Willie et al. (2023) | Staff members at community healthcare clinics involved in PrEP clinical services (7) | 6 women (85.7%) | 7 African American (100%) | NR | USA | Semi-structured interview | Thematic analysis |
| McGeown et al. (2023) | Researcher, academic general practitioners, and staff at support charity for marginalized women (5) | 5 women (100%) | NR | NR | United Kingdom | Semi-structured interview | Framework approach |
| Giesbrecht et al. (2023) | Service providers who work with newcomer women victim-survivors (32) | 32 women (100%) | 14 had migrated to Canada from another country (44%) | 34% less than 5 years; 34% 5–10 years; 22% 10–24 years; 6% 25–34 years; 3% over 35 years | Canada | Focus group | Thematic analysis |
| Wekerle et al. (2022) | Indigenous Canadian health care and social service providers (21) | 14 women (66.7%) | 21 Indigenous Canadian (100%) | 4 participants reported “all my life”; range of remainder 0–30 years | Canada | Semi-structured interview | Inductive content analysis |
| van der Hoeven et al. (2022) | Crisis shelter professionals (18) | 17 women (100%) | Country of birth:  16 Canada (94.1%)  1 Poland (5.9%) | Mean: 4 years’ experience with program; range 0.5–9 years | Netherlands | Semi-structured interview | Codebook thematic analysis |
| Wood et al. (2022) | Hotline staff involved in chat/text advocacy (11) | 11 women (100%) | 8 Hispanic and/or Indigenous American (72.7%)  3 White (27.2%) | NR | USA | Semi-structured interview; de-identified transcripts | Grounded theory approach; content analysis |
| Ghidei et al. (2022) | Service providers within the health and anti-violence sectors (24) | 17 women (70.8%) | 14 white (58.3%)  7 non-white (Indigenous, Black or South Asian, 29.1%) | Majority (n=15) < 10 years | Canada | Semi-structured interview | Inductive thematic analysis |
| Armstrong (2022) | Practitioners, policymakers, and researchers involved in work on DV, SUD, or both (27) | NR | NR | Median 27 years; range 9 to 47 years | USA | Semi-structured interview | Grounded theory analysis |
| McConnell & Phelan (2022) | Key informants who have experience supporting women with intellectual disability affected by DV (5) | 5 women (100%) | NR | Range 3–25 years | Canada | Interview | Qualitative descriptive analysis |
| Ahrens et al. (2022) | Staff who worked closely with Vietnamese-heritage survivors (13) | NR | “Most (but not all) of Vietnamese descent” | NR | USA | Focus group | Analytic Induction |
| Tarshis et al. (2022) | Service providers from a variety of DV organizations (10) | 10 women (100%) | 3 White (30%)  3 Asian (30%)  3 Black/African American (30%)  1 Other/Mixed (10%) | Mean= 10 years; range 3–10 years | USA | Semi-structured interview | Grounded theory and constructivist approach |
| Morton et al. (2021) | Practitioners within a DV service (6) | NR | NR | NR | Ireland | Written narrative | Narrative |
| Dawson et al. (2021) | Counselling and mental health practitioners at a shelter (7) | NR | NR | NR | Australia | Semi-structured interview | Inductive thematic analysis |
| Wood et al. (2021) | Campus-based advocates (23) | 19 women (82.6%) | 12 White (52.2%)  10 Non-White (43.5%) | NR | USA | Semi-structured interview | Thematic analysis |
| Anderson et al. (2021) | Service providers who interact with high school and/or college students who have experienced sexual assault or DV (6) | 6 women (100%) | 5 White (83%)  1 Mixed/Other (17%) | NR | USA | Semi-structured interview | Thematic analysis |
| Kahan et al. (2020) | Shelter staff and peer mentors (7) | NR | NR | NR | Canada | Interview | Thematic analysis |
| Désilets et al. (2020) | HIV and DV community service providers (12) | 10 women (83%) | 7 Canadian (58.3%)  2 Caribbean (16.7%)  1 First Nation (8.3%)  1 Sub-Saharan African (8.3%)  1 Western European (8.3%) | Mean 7 years in current position | Canada | Focus group | Direct content analysis |
| Hegarty et al. (2020) | Midwives or doctors working in an antenatal clinic or social workers at sites providing pregnancy care (91) | NR | NR | NR | Australia | Focus group; interview | Framework analysis |
| Bailey et al. (2020) | Practitioners from substance use, DV, and women’s specialist criminal justice services (14) | NR | NR | NR | United Kingdom | Semi-structured interview | Thematic analysis using a pragmatic approach |
| Mengo et al. (2020) | Staff and administrators in DV advocacy agencies and shelters (62) | NR | NR | NR | USA | Focus group | Structural coding and thematic development |
| Agu et al. (2020) | Home visitors for pregnant women | NR | NR |  | USA | Online survey | Thematic content analysis |
| Kulkarni et al. (2019) | DV shelter directors and coalition leaders (7) | NR | NR | NR | USA | Semi-structured interview | Thematic analysis |
| Williams et al. (2017) | IPV and HIV service providers (9) | 9 women (100%) | 1 Black (11.1%)  6 Hispanic (66.7%)  2 White (22.2%) | Majority (5) more than 1 year (55.6%) | USA | Semi-structured interview | Direct content analysis |
| Ward-Lasher et al. (2017) | Employees of a trauma-informed housing agency (7) | 7 women (100%) | NR | Mean 4 years | USA | Semi-structured interview | Coded analysis |
| Miller et al. (2017) | Health care providers and administrators at family planning clinics (23) | 23 women (100%) | NR | Two-thirds > 10 years | USA | Semi-structured interview | Consensus coding approach |
| Simmons et al. (2016) | Helping professionals working in DV programs (187) | 180 women (96.2%) | 13 African American (6.9%)  155 Caucasian (82.9%)  9 Hispanic (4.8%)  10 Other (5.3%) | Mean 12.24 years; range 0–37 years | USA | Online survey | Concept mapping approach |
| Goodman et al. (2016) | DV shelter executive directors, shelter directors, and direct service coordinators (28) | 28 women (100%) | 27 White (96.4%)  1 Indigenous American (3.5%) | Mean 12.9 years; range 0–35 years | USA | Focus group | Content analysis |

Note. Studies are listed chronologically by date of publication. NR: not recorded. USA: United States of America. DV: domestic violence. SUD: substance use disorders. HIV: human immunodeficiency virus. PrEP: pre-exposure prophylaxis.

**Table B2**

*Quality Assessment of Included Studies*

| Authors (Year) | 1 | 2 | 3 | 4 | 5 | 6 | 7 | 8 | 9 | 10 | Quality |
| --- | --- | --- | --- | --- | --- | --- | --- | --- | --- | --- | --- |
| Jolof et al. (2024) | + | + | + | + | + | + | + | + | + | + | High |
| Willie et al. (2023) | + | + | + | + | + | + | + | + | + | + | High |
| McGeown et al. (2023) | + | + | + | + | + | + | + | + | + | + | High |
| Giesbrecht et al. (2023) | + | + | + | + | + | - | + | + | + | + | High |
| Wekerle et al. (2022) | + | + | + | + | + | + | + | + | + | + | High |
| van der Hoeven et al. (2022) | + | + | - | - | + | - | - | + | + | ? | Low |
| Wood et al. (2022) | + | + | + | + | + | + | + | + | + | + | High |
| Ghidei et al. (2022) | + | + | + | + | + | + | + | + | + | + | High |
| Armstrong (2022) | + | + | + | + | + | + | + | + | + | + | High |
| McConnell & Phelan (2022) | + | + | + | – | + | – | + | – | + | ? | Low |
| Ahrens et al. (2022) | + | + | + | + | + | + | + | + | + | + | High |
| Tashis et al. (2022) | + | + | + | + | + | + | + | + | + | + | Moderate |
| Morton et al. (2021) | + | ? | ? | ? | – | – | **–** | – | – | + | Low |
| Dawson et al. (2021) | + | + | + | - | + | - | **-** | + | + | - | Low |
| Wood et al. (2021) | + | + | + | + | + | + | + | + | + | + | High |
| Anderson et al. (2021) | + | + | + | - | + | - | - | - | + | + | Moderate |
| Kahan et al. (2020) | + | + | + | + | + | - | + | + | + | + | High |
| Désilets et al. (2020) | + | + | + | + | + | + | + | + | + | + | High |
| Hegarty et al. (2020) | + | + | + | + | + | + | + | + | + | + | High |
| Bailey et al. (2020) | + | + | + | + | + | + | + | + | + | + | High |
| Mengo et al. (2020) | + | + | + | + | + | + | + | + | + | ? | Moderate |
| Agu et al. (2020) | + | + | + | + | + | - | + | + | + | + |  |
| Kulkarni et al. (2019) | + | + | + | ? | + | – | – | + | + | + | Moderate |
| Williams et al. (2017) | + | + | + | + | + | + | + | + | + | + | High |
| Ward-Lasher et al. (2017) | + | + | + | ? | ? | – | – | – | + | + | Low |
| Miller et al. (2017) | + | + | + | + | + | + | + | + | + | + | High |
| Simmons et al. (2016) | + | + | ? | + | + | + | – | + | + | + | Moderate |
| Goodman et al. (2016)s | + | + | + | + | + | - | ? | + | + | + | Moderate |

*Note.* Studies are listed chronologically by date of publication. Criteria: 1, Was there a clear statement of the aims of the research? 2, Is a qualitative methodology appropriate? 3, Was the research design appropriate to address the aims of the research? 4, Was the recruitment strategy appropriate to the aims of the research? 5, Was the data collected in a way that addressed the research issue? 6, Has the relationship between researcher and participants been adequately considered? 7, Have ethical issues been taken into consideration? 8, Was the data analysis sufficiently rigorous? 9, Is there a clear statement of findings? 10, How valuable is the research? Symbols: +, yes; –, no; ?, cannot determine or not reported.

**Table B3**

*Themes Derived from the Included Studies*

| Theme/Subtheme | Description | No. of articles | Article Citations |
| --- | --- | --- | --- |
| *Individual-Level Themes* | | | |
| 1. **Safety** | | | |
| a. Safe and secure support | Effective support centers safety through safe relationships with victim-survivors, secure physical environments, and addressing immediate safety concerns. | 22 | Agu et al. (2020) et al. (2020); Ahrens et al. (2022) et al.; Armstrong (2023); Bailey et al. (2020) et al.; Dawson et al. (2021) et al.; Désilets et al. (2020) et al.; Giesbrecht et al. (2023); Goodman et al. (2016); Hegarty et al. (2020); Van der Hoeven et al. (2023); Kahan et al. (2020); Kulkarni et al. (2019); McConnell & Phelan (2022); McGeown et al. (2023); Mengo et al. (2020); Morton et al. (2021); Ward-Lasher et al. (2017); Wekerle et al. (2022); Williams et al. (2017); Willie et al. (2023); Wood et al. (2022); Wood et al. (2021) |
| b. Trauma awareness for harm reduction | Trauma-informed practices involve incorporating a sensitivity to the impacts of trauma and ensuring privacy and confidentiality are essential in order to reduce the potential for harm and retraumatization. | 23 | Agu et al. (2020); Ahrens et al. (2022); Anderson et al. (2021); Armstrong (2023); Bailey et al. (2020); Désilets et al. (2020); Ghidei et al. (2022); Giesbrecht et al. (2023); Goodman et al. (2016); Hegarty et al. (2020); Jolof et al. (2024); Kahan et al. (2020); Kulkarni et al. (2019); McConnell & Phelan (2022); McGeown et al. (2023); Mengo et al. (2020); Morton et al. (2021); Simmons et al. (2017); Tarshis et al. (2022); Ward-Lasher et al. (2017); Williams et al. (2017); Wood et al. (2022); Wood et al. (2021) |
| 2. **Survivor-centered care** | | | |
| a. Information and education | Providing accurate, accessible information and psychoeducation empowers victim-survivors to make informed choices and center their needs. | 18 | Agu et al. (2020); Désilets et al. (2020); Geisbrecht; Hegarty et al. (2020); Wood et al. (2022); Bailey et al. (2020); Kahan et al. (2020); Wood et al. (2021); Morton et al. (2021); Simmons et al. (2017); Ghidei et al. (2022); Goodman et al. (2016); McConnell & Phelan (2022); Miller et al. (2017); Ward-Lasher et al. (2017); Williams et al. (2017); Willie et al. (2023) |
| b. Provision of relevant resources | Matching victim-survivor needs to resources and guiding them through navigation of services are considered key roles for service providers. | 16 | Agu et al. (2020); Armstrong (2023); Bailey et al. (2020); Désilets et al. (2020); Giesbrecht et al. (2023); Goodman et al. (2016); Hegarty et al. (2020); Jolof et al. (2024); Kulkarni et al. (2019); McConnell & Phelan (2022); Simmons et al. (2017); Ward-Lasher et al. (2017); Williams et al. (2017); Willie et al. (2023); Wood et al. (2021); Wood et al. (2022) |
| c. Catering to unique, individual needs | Service providers adapt and individualize care to meet the unique needs of victim-survivors, involving flexibility and creativity. | 20 | Ahrens et al. (2022); Anderson et al. (2021); Dawson et al. (2021); Désilets et al. (2020); Ghidei et al. (2022); Giesbrecht et al. (2023); Jolof et al. (2024); Kahan et al. (2020); McConnell & Phelan (2022); Mengo et al. (2020); Wekerle et al. (2022); Bailey et al. (2020); Hegarty et al. (2020); Wood et al. (2022); Tarshis et al. (2022); Goodman et al. (2016); Kulkarni et al. (2019); Williams et al. (2017); Ward-Lasher et al. (2017); Wood et al. (2021) |
| 3. **Respectful emotional support** | | | |
| a. Empathetic support | Effective care involves treating victim-survivors with empathy and respect through empathic communication, respecting their preferences and needs, and respectful listening. | 24 | Agu et al. (2020); Ahrens et al. (2022); Anderson et al. (2021); Armstrong (2023); Bailey et al. (2020); Dawson et al. (2021); Désilets et al. (2020); Ghidei et al. (2022); Giesbrecht et al. (2023); Goodman et al. (2016); Hegarty et al. (2020); Kahan et al. (2020); Jolof et al. (2024); Kulkarni et al. (2019); McConnell & Phelan (2022); McGeown et al. (2023); Mengo et al. (2020); Miller et al. (2017); Morton et al. (2021); Tarshis et al. (2022); Ward-Lasher et al. (2017); Williams et al. (2017); Wood et al. (2022); Wood et al. (2021) |
| b. Compassion and validation | Practitioners provide effective emotional support using compassion, validation, and a caring approach. | 16 | Ahrens et al. (2022); Anderson et al. (2021); Dawson et al. (2021); Désilets et al. (2020); Giesbrecht et al. (2023); Goodman et al. (2016); Hegarty et al. (2020); Kahan et al. (2020); Kulkarni et al. (2019); McGeown et al. (2023); Morton et al. (2021); Simmons et al. (2017); Ward-Lasher et al. (2017); Williams et al. (2017); Wood et al. (2022); Wood et al. (2021) |
| c. Trauma-focused mental health support | Due to the high prevalence of mental health concerns, victim-survivor mental health needs to be addressed using trauma-focused interventions and supporting the development of coping and emotion regulation strategies. | 16 | Armstrong (2023); Bailey et al. (2020); Dawson et al. (2021); Giesbrecht et al. (2023); Kahan et al. (2020); Jolof et al (2024); Kulkarni et al. (2019); McConnell & Phelan (2022); Mengo et al. (2020); Morton et al. (2021); Simmons et al. (2017); Tarshis et al. (2022); Van der Hoeven et al. (2023); Ward-Lasher et al. (2017); Wood et al. (2021); Wood et al. (2022) |
| 4. **Non-judgmental, reciprocal client-practitioner relationship** | | | |
| a. Mutual relationship | Service providers encourage equal, collaborative relationships between practitioners and victim-survivors which emphasize the lived experience of victim-survivors, incorporating transparency and considering boundaries. | 22 | Ahrens et al. (2022); Armstrong (2023); Bailey et al. (2020); Dawson et al. (2021); Désilets et al. (2020); Ghidei et al. (2022); Giesbrecht et al. (2023); Goodman et al. (2016); Hegarty et al. (2020); Jolof et al (2024); Kahan et al. (2020); Kulkarni et al. (2019); McConnell & Phelan (2022); McGeown et al (2023); Miller et al. (2017); Morton et al. (2021); Simmons et al. (2017); Tarshis et al. (2022); Van der Hoeven et al. (2023); Ward-Lasher et al. (2017); Wood et al. (2022); Wood et al. (2021) |
| b. Strengths-based approach | The importance of a non-judgmental and non-pathologizing approach which emphasizes the resilience and coping abilities of victim-survivors, and preventing stigmatization, over a traditional medical model. | 18 | Agu et al. (2020); Anderson et al. (2021); Armstrong (2023); Bailey et al. (2020); Hegarty et al. (2020); Kulkarni et al. (2019); McConnell & Phelan (2022); McGeown et al (2023); Mengo et al. (2020); Morton et al. (2021); Simmons et al. (2017); Tarshis et al. (2022); Van der Hoeven et al. (2023); Ward-Lasher et al. (2017); Wekerle et al. (2022); Williams et al. (2017); Wood et al. (2022); Wood et al. (2021) |
| 5. **Supporting victim-survivors to thrive** | | | |
| a. Supporting healthy social connections | Service providers help victim-survivors to develop healthy social connections, navigate potential challenges, and assess safety in relationships. | 12 | Ahrens et al. (2022); Armstrong (2023); Bailey et al. (2020); Goodman et al. (2016); Kahan et al. (2020); Kulkarni et al. (2019); McConnell & Phelan (2022); McGeown et al (2023); Van der Hoeven et al. (2023); Ward-Lasher et al. (2017); Wekerle et al. (2022); Williams et al. (2017); Wood et al. (2022); Wood et al. (2021) |
| b. Promoting agency and sense of self | Practitioners empower victim-survivors through supporting their independence, autonomy, and choice, and affirming their identities in order to develop a more positive sense of self. | 21 | Agu et al. (2020); Ahrens et al. (2022); Anderson et al. (2021); Bailey et al. (2020); Dawson et al. (2021); Désilets et al. (2020); Ghidei et al. (2022); Giesbrecht et al. (2023); Goodman et al. (2016); Hegarty et al. (2020); Kahan et al. (2020); Kulkarni et al. (2019); McConnell & Phelan (2022); McGeown et al (2023); Mengo et al. (2020); Morton et al. (2021); Simmons et al. (2017); Tarshis et al. (2022); Ward-Lasher et al. (2017); Wood et al. (2022); Wood et al. (2021) |
| c. Rebuilding and improving victim-survivor lives | Recovery involves supporting victim-survivors to rebuild their lives and find meaning, hope, and enjoyment through engaging in enjoyed activities and reconnecting with community. | 8 | Goodman et al. (2016); Van der Hoeven et al. (2023); Kulkarni et al. (2019); McConnell & Phelan (2022); Mengo et al. (2020); Morton et al. (2021); Simmons et al. (2017); Wood et al. (2021) |
| 6. **Cultural safety and inclusivity** | | | |
| a. Intersectionality | Service providers recognize the complex realities of victim-survivors’ lives and incorporate an awareness of the structural factors and intersecting types of oppressions that victim-survivors experience. | 20 | Agu et al. (2020); Armstrong (2023); Bailey et al. (2020); Dawson et al. (2021); Désilets et al. (2020); Ghidei et al. (2022); Giesbrecht et al. (2023); Hegarty et al. (2020); Jolof et al. (2024); Kulkarni et al. (2019); McConnell & Phelan (2022); McGeown et al. (2023); Mengo et al. (2020); Morton et al. (2021); Tarshis et al. (2022); Ward-Lasher et al. (2017); Wekerle et al. (2022); Williams et al. (2017); Willie et al. (2023); Wood et al. (2021) |
| b. Inclusive practice | Inclusivity is essential to provide services that are accessible to everyone and meet the needs of victim-survivors from all backgrounds and circumstances. | 20 | Ahrens et al. (2022); Anderson et al. (2021); Bailey et al. (2020); Dawson et al. (2021); Désilets et al. (2020); Ghidei et al. (2022); Giesbrecht et al. (2023); Goodman et al. (2016); Hegarty et al. (2020); Jolof et al. (2024); Kahan et al. (2020); Kulkarni et al. (2019); McConnell & Phelan (2022); Mengo et al. (2020); Morton et al. (2021); Van der Hoeven et al. (2023); Wekerle et al. (2022); Williams et al. (2017); Wood et al. (2022); Wood et al. (2021) |
| c. Cultural safety | The importance of cultural safety and awareness, respecting culture and tradition, incorporating different cultural norms and values into support, and engaging in culturally relevant practices. | 14 | Agu et al. (2020); Ahrens et al. (2022); Anderson et al. (2021); Dawson et al. (2021); Désilets et al. (2020); Ghidei et al. (2022); Giesbrecht et al. (2023); Goodman et al. (2016); Kulkarni et al. (2019); McConnell & Phelan (2022); Morton et al. (2021); Tarshis et al. (2022); Wekerle et al. (2022); Wood et al. (2022) |
| *System-Level Themes* | | | |
| 7. **Provision of effective and accessible services** | | | |
| a. Flexible services with reduced complexity | Services that are timely, easily accessible, and flexible in timing and requirements overcome barriers to engagement. | 11 | Ahrens et al. (2022); Anderson et al. (2021); Bailey et al. (2020); Hegarty et al. (2020); Kahan et al. (2020); Kulkarni et al. (2019); McGeown et al. (2023); Mengo et al. (2020); Morton et al. (2021); Wood et al. (2022); Wood et al. (2021) |
| b. Continuity of care | Providing continuity of care is important to avoid retraumatization and promote service engagement. | 9 | Ahrens et al. (2022); Goodman et al. (2016); Hegarty et al. (2020); Jolof et al. (2024); Tarshis et al. (2022); Ward-Lasher et al. (2017); Williams et al. (2017); Wood et al. (2022); Wood et al. (2021) |
| c. Inclusive services | Service providers described system-level practices to promote inclusivity and meet the needs of all victim-survivors. | 12 | Ahrens et al. (2022); Anderson et al. (2021); Dawson et al. (2021); Ghidei et al. (2022); Giesbrecht et al. (2023); Hegarty et al. (2020); Jolof et al. (2024); Kulkarni et al. (2019); Van der Hoeven et al. (2023); Wekerle et al. (2022) |
| d. Service improvement strategies | Services can be improved through providing adequate support and training for staff and embodying a trauma-and-violence-informed approach to organizational culture at all service levels. | 26 | Agu et al. (2020); Ahrens et al. (2022); Armstrong (2023); Bailey et al. (2020); Dawson et al. (2021); Désilets et al. (2020); Ghidei et al. (2022); Giesbrecht et al. (2023); Goodman et al. (2016); Hegarty et al. (2020); Jolof et al. (2024); Kahan et al. (2020); Kulkarni et al. (2019); McConnell & Phelan (2022); McGeown et al. (2023); Mengo et al. (2020); Miller et al. (2017); Morton et al. (2021); Simmons et al. (2017); Tarshis et al. (2022); Ward-Lasher et al. (2017); Wekerle et al. (2022); Williams et al. (2017); Willie et al. (2023); Wood et al. (2022); Wood et al. (2021) |
| 8. **Providing holistic services** | | | |
| a. Comprehensively meeting needs | A holistic approach to service provision is necessary to comprehensively address victim-survivor needs and promote recovery, through collaboration between services and supporting basic needs. | 22 | Agu et al. (2020); Ahrens et al. (2022); Armstrong (2023); Désilets et al. (2020); Ghidei et al. (2022); Giesbrecht et al. (2023); Goodman et al. (2016); Hegarty et al. (2020); Jolof et al. (2024); Kahan et al. (2020); Kulkarni et al. (2019); McConnell & Phelan (2022); McGeown et al. (2023); Mengo et al. (2020); Miller et al. (2017); Morton et al. (2021); Simmons et al. (2017); Tarshis et al. (2022); Ward-Lasher et al. (2017); Williams et al. (2017); Wood et al. (2022); Wood et al. (2021) |
| b. Community engagement and prevention | Services extend beyond the individual to address DFSV at the community level and facilitate help-seeking through raising awareness and community engagement. | 8 | Anderson et al. (2021); Désilets et al. (2020); Miller et al. (2017); Wekerle et al. (2022); Williams et al. (2017); Willie et al. (2023); Wood et al. (2022); Wood et al. (2021) |

*Note.* DV: domestic, family, and sexual violence.
